# Expression in *Escherichia coli*, purification, refolding and antifungal activity of an osmotin from *Solanum nigrum*

**DOI:** 10.1186/1475-2859-7-7

**Published:** 2008-03-11

**Authors:** Magnólia de A Campos, Marilia S Silva, Cláudio P Magalhães, Simone G Ribeiro, Rafael PD Sarto, Eduardo A Vieira, Maria F Grossi de Sá

**Affiliations:** 1 EMBRAPA Recursos Genéticos e Biotecnologia, PO Box 02372, 70770-900, Brasília-DF, Brazil; 2 Departamento de Biologia Celular, Universidade de Brasília, PO Box 90710-900, Brasília-DF, Brazil; 3 EMBRAPA Cerrados, BR 020 Km 18, PO Box 08223, 73310-970, Planaltina-DF, Brazil; 4 Departamento de Biologia, Universidade Federal de Lavras, P.O.Box 3037, 37200-000, Lavras-MG, Brazil

## Abstract

**Background:**

Heterologous protein expression in microorganisms may contribute to identify and demonstrate antifungal activity of novel proteins. The *Solanum nigrum *osmotin-like protein (SnOLP) gene encodes a member of pathogenesis-related (PR) proteins, from the PR-5 sub-group, the last comprising several proteins with different functions, including antifungal activity. Based on deduced amino acid sequence of SnOLP, computer modeling produced a tertiary structure which is indicative of antifungal activity.

**Results:**

To validate the potential antifungal activity of SnOLP, a hexahistidine-tagged mature SnOLP form was overexpressed in *Escherichia coli *M15 strain carried out by a pQE30 vector construction. The urea solubilized His_6_-tagged mature SnOLP protein was affinity-purified by immobilized-metal (Ni^2+^) affinity column chromatography. As SnOLP requires the correct formation of eight disulfide bonds, not correctly formed in bacterial cells, we adapted an *in vitro *method to refold the *E. coli *expressed SnOLP by using reduced:oxidized gluthatione redox buffer. This method generated biologically active conformations of the recombinant mature SnOLP, which exerted antifungal action towards plant pathogenic fungi (*Fusarium solani *f. sp.*glycines*, *Colletotrichum *spp., *Macrophomina phaseolina*) and oomycete (*Phytophthora nicotiana var. parasitica*) under *in vitro *conditions.

**Conclusion:**

Since SnOLP displays activity against economically important plant pathogenic fungi and oomycete, it represents a novel PR-5 protein with promising utility for biotechnological applications.

## Background

Plants have evolved a complex array of chemical and enzymatic defenses, both constitutive and inducible, which are not involved in pathogen detection but whose effectiveness influences pathogenesis and disease resistance [1]. Plants protect themselves from pathogen invasion through the local expression of a variety of cysteine-rich antimicrobial peptides and a set of pathogenesis-related (PR) proteins [2,3]. Interestingly, most of the components belonging to these two classes of defense proteins are antifungal proteins, even though they are highly divergent in primary structure, in length and exhibit different direct antimicrobial activity.

The family of PR-5 proteins (also known as permatins, thaumatins or osmotins) is part of a larger group of proteins so-called PR-proteins, the last being classified into 17 families (PR-1 to PR-17) [3-5]. Neutral, basic and acid isoforms of PR-5 proteins have been found in plants, all of them consisting of cysteine-rich proteins involved in plant defense responses to several pathogens and abiotic stresses. Many PR-5 genes are activated by different signals such as abscisic acid, ethylene, auxin, salinity, lack of water, cold, UV light, wounding, virus and fungal/oomycete infection, that result in PR-5 protein accumulation in plant cells [6-8].

Different activities have been ascribed to the members of this family [9-13], especially antifungal/antioomycetal *in vitro *and *in planta *activity for most of them [14,15]. It is not known how PR-5 proteins exert the antifungal activity demonstrated through *in vitro *inhibition of hyphal growth and spore germination, spore lysis and reduction in viability of germinated spores. It has been proposed that they may act by permeabilization of fungal membranes or interaction with fungal membrane receptors [13,15-19]. In addition, it has been demonstrated that a number of PR-5 proteins bind β-1,3-glucan and have detectable *in vitro *β-1,3-glucanase activity [20,21]. Moreover, a tobacco osmotin induces apoptosis in *Saccharomyces cerevisiae *[22]. Nevertheless, the molecular mechanisms of membrane permeabilization, interaction with fungal receptor or apoptosis remain not completely understood.

Intensive efforts have been undertaken to find *PR-5 *genes encoding for novel putative antifungal proteins, which could be used in agricultural and/or pharmaceutical biotechnological approaches to control fungal diseases. In this context and based on previously published evidences, wild *Solanum *plant species represent a valuable source of natural plant resistance against many fungi and oomycetes, in which PR-5 proteins might be involved [23-26]. However, in order to evaluate the potential of these proteins as source of plant resistance, their ability to display antifungal activity needs to be proven experimentally. In order to test and characterize the *in vitro *activity of a particular protein, the first step is to purify the functional protein in large scale [27,28].

Supporting these claims, the majority of PR-5 proteins have been purified from plant native conditions. The demonstration of the antifungal activity of a PR-5 protein predicted from gene sequence requires its *in vivo *(cell system or *in planta*) expression by and purification from a heterologous systems [13,29-31]. In a previous work, we have described the isolation and cloning of a gene (*SnOLP*) coding for a neutral osmotin-like protein from *Solanum nigrum *var. *americanum *[32]. Based on deduced amino acid sequence of the SnOLP protein, a computer modeling produced a structure that is indicative of antifungal activity. Herein, it is described the validation of SnOLP activity against plant pathogenic fungi and oomycete. This validation was performed by expression of the *SnOLP *gene in *Escherichia coli*, followed by purification of mature SnOLP and subsequent *in vitro *refolding, what generated biologically active conformations of the protein.

## Results

### Expression and purification of His_6_-tagged mature SnOLP

**Figure 1:**
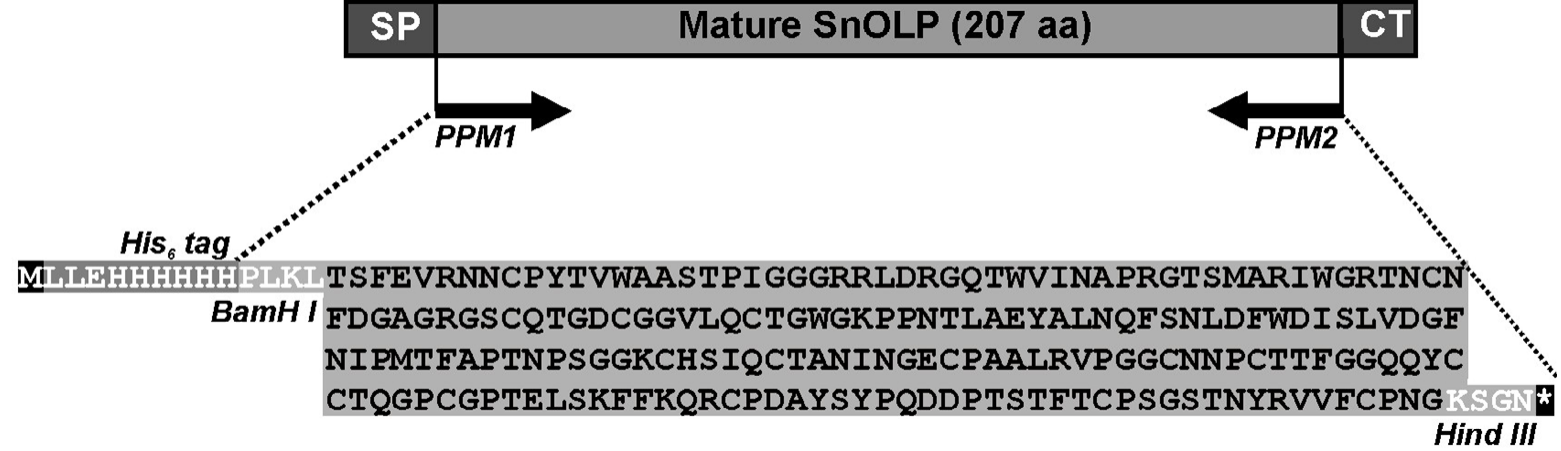
**Schematic description of the construct designed to express His_6_-tagged mature SnOLP in *E. coli***. The regions of the preproprotein SnOLP are shown. The positions of the primers used to generate fragments coding for mature SnOLP form are shown. The orientations of the primers **PPM1 **and **PPM2**, including the respective restriction cloning sites, are indicated by arrows. The His_6_-tag encoded by the pQE30 vector is indicated. **SP**, Signal Peptide; **CT**, Carboxy-Terminal propeptide; **M**, Methionine; *, Stop codon.

The hexahistidine (His_6_)-tagged mature form of a neutral osmotin-like protein from *Solanun nigrum *L. var. *americanum *(SnOLP) was overexpressed in *E. coli *heterologous system by using pQE30 expression vector and purified by using immobilized-metal (Ni^2+^) affinity chromatography (IMAC). Since bacterial expression systems do not perform certain post-translational processing, deletion mutations were generated by PCR amplification from the previously cloned *SnOLP *gene [32] to produce a mature SnOLP form lacking its signal peptide and its carboxy-terminal peptide. This PCR product was cloned into the pQE30 vector, which contains an inbuilt His_6_-tag sequence, what resulted into a His_6_-tagged mature SnOLP coding sequence (pQE30-SnOLP construct, Fig. 1). The expected His_6_-tagged mature SnOLP protein is 225 amino acids in length with a theoretical *M*_*r *_of 24,363 KDa, calculated from deduced amino acid sequence (ExPASY Protein Parameters Tools Analysis).

**Figure 2:**
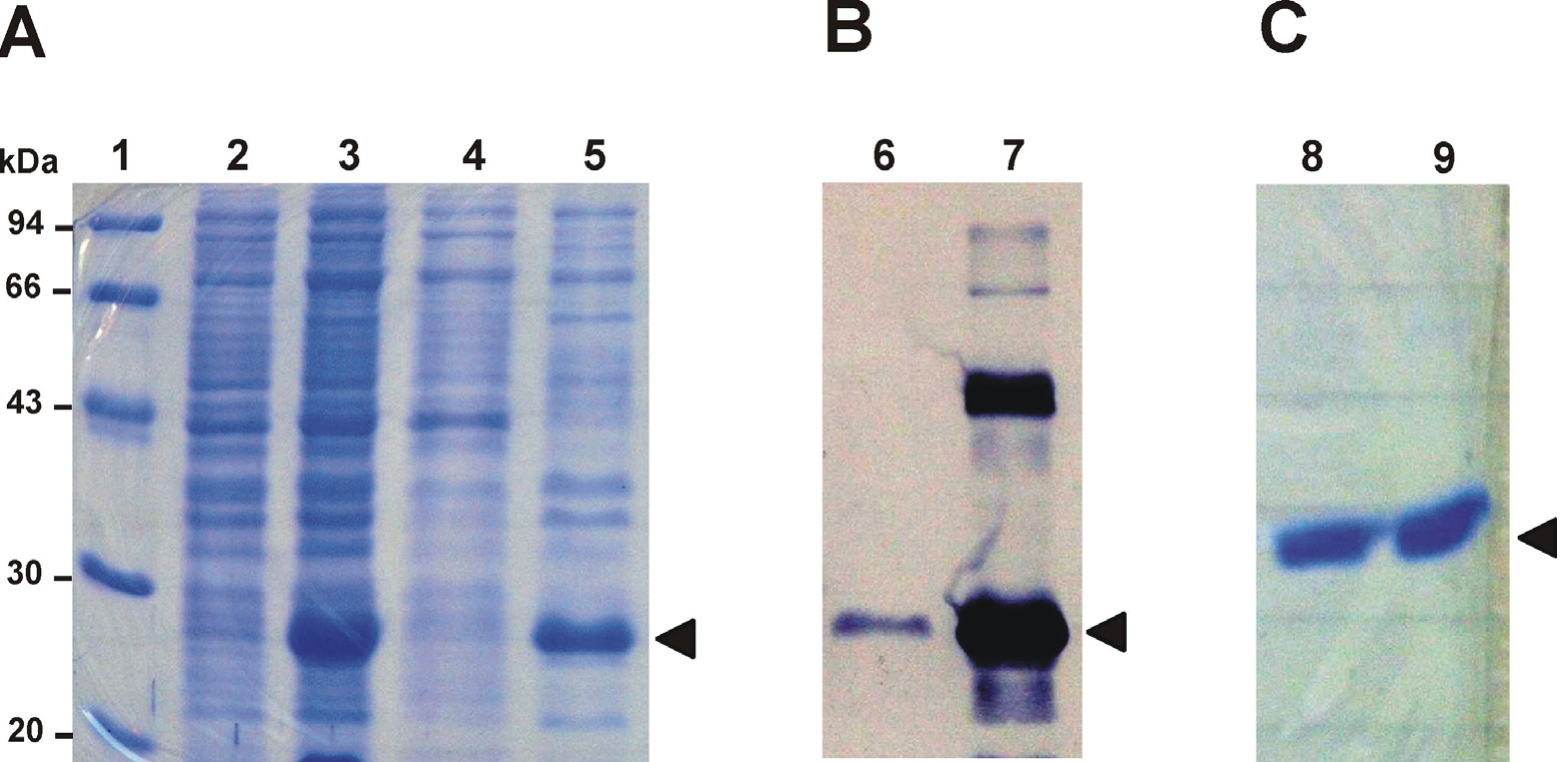
**Expression of His_6_-tagged mature SnOLP in *E. coli*, solubilization and purification**. **A**. SDS-PAGE analysis of the expression of His_6_-tagged mature SnOLP in *E. coli *cultures incubated at 37°C for 3 h and induced by 0.4 mM IPTG (when indicated). **1**. Molecular mass marker (LMW, Amersham Pharmacia Biotech); **2**. Total protein fraction from non-induced *E. coli *culture; **3**. Total protein fraction from IPTG-induced *E. coli *culture; **4**. Soluble protein fraction from IPTG-induced *E. coli *culture; **5**. Insoluble protein fraction from IPTG-induced *E. coli *culture. **B**. Western blot analysis of expressed His_6_-tagged mature SnOLP probed with His_6_-monoclonal antibody. **6**. Soluble protein fraction from IPTG-induced *E. coli *culture; **7**. Insoluble protein fraction from IPTG-induced *E. coli *culture. **C**. SDS-PAGE analysis of insoluble His_6_-tagged mature SnOLP which was urea solubilized and subsequently purified by immobilized-metal (Ni^2+^) affinity chromatography (IMAC). **8 **and **9**. Eluates from IMAC. In **A**. and **C**. the gels were stained with Coomassie brilliant blue. His_6_-tagged mature SnOLP protein is indicated by arrow heads.

In order to obtain information on the solubility of bacterially produced His_6_-tagged mature SnOLP, this protein was expressed in IPTG-induced M15 *E. coli *cells carrying the pQE30-SnOLP construct. Total cell protein fraction was obtained from non-induced and induced bacterial cells lysed under native conditions. Then soluble and insoluble protein fractions from induced protein fractions were separated by centrifugation. Equal amounts of total, soluble and insoluble protein fractions were analyzed on 12% SDS-PAGE (Fig. 2A). A protein presenting a mass around the predicted *M*_*r *_of the His_6_-tagged mature SnOLP was present in high amounts within the induced total protein fraction as compared to the non-induced total protein fraction (Fig. 2A, lanes 2 and 3). The majority of the probable His_6_-tagged mature SnOLP is present within the insoluble protein fraction, as compared to the soluble protein fraction (Fig. 2A, lanes 4 and 5), and represents around a third of the total protein fraction contents. As confirmed by Western blotting, low quantity of the His_6_-tagged mature SnOLP is present within the soluble fraction whereas high quantity is detected within the insoluble fraction (Fig. 2B, lanes 6 and 7). Curiously, an extra band, higher than the putative His_6_-tagged mature SnOLP, is present within the insoluble fraction though absent in the soluble fraction (Fig. 2B, lanes 6 and 7). This extra band may be due to dimeric forms of the His_6_-tagged mature SnOLP.

Supported by these findings, an expression in a larger scale, in order to purify His_6_-tagged mature SnOLP, was performed by *E. coli *culture incubated at 37°C for 2 h after expression induction by IPTG 0.4 mM. This larger scale expression protocol produced high quantities of insoluble His_6_-tagged mature SnOLP, which was subsequently urea-solubilized and purified by IMAC under denaturing conditions. SDS-PAGE analysis of IMAC eluates revealed that purification of the bacterially expressed and solubilized His_6_-tagged mature SnOLP provided virtually 100% pure protein, as evident by the single band detected in two representative IMAC eluates (Fig. 2C, lanes 8 and 9). These procedures led to elevated yields of high quality pure and soluble, though denatured, His_6_-tagged mature SnOLP (~1 mg/mL) from 500 mL of induced *E. coli *culture. In order to promote the recovery of steric structures and biological activity of the pure soluble denatured His_6_-tagged mature SnOLP, the fusion protein was slowly renatured in buffer containing a redox state maintained by reduced-glutathione:oxidized-glutathione pair, followed by dialysis against water. No significant precipitation was observed during or after refolding.

### *In vitro *antifungal activity studies using His_6_-tagged mature SnOLP

**Figure 3:**
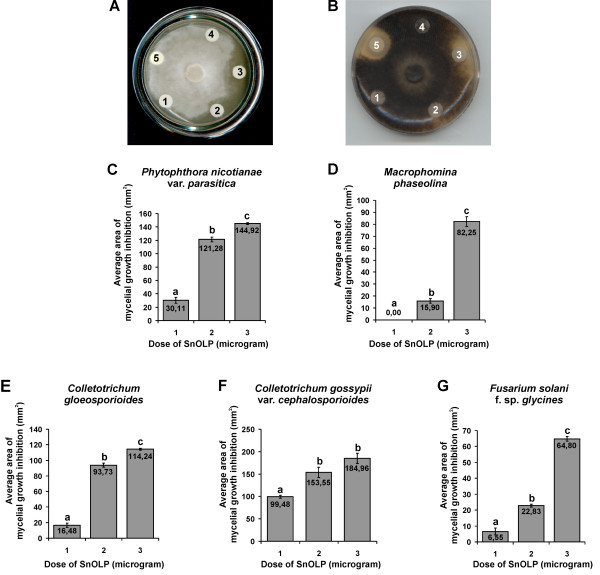
**Bioassays of the mycelial growth inhibition activity of purified and renatured His_6_-tagged mature SnOLP**. **A-B**. Representative bioassay Petri dishes. Filter paper discs containing 10 μL of test proteins were placed on plates 3 days after inoculation with fungal mycelia (plugs). Petri dishes were incubated at 29°C during the entire bioassay. Inhibitory effects of purified and renatured His_6_-tagged mature SnOLP upon the fungi are observed as areas lacking mycelial growth. **A**. *Phytophthora nicotiana *var. *parasitica*; 1 day after adding protein (a.a.p.); **B**. *Macrophomina phaseolina*, 5 days a.a.p. **1–3**. Corresponds to the doses of 1 μg, 2 μg and 3 μg of purified and renatured His_6_-tagged mature SnOLP, or to the concentrations of 0,1 μg/μL, 0,2 μg/μL and 0,3 μg/μL, respectively; **4**. Corresponds to the dose of 10 μg of Bovine Serum Albumin (BSA), or to the concentration of 1 μg/μL; **5**. Corresponds to the dose of 2000 U Nistatin, or to the concentration of 200 U/μL. **C-F**. Average area (mm^2^) of mycelial growth inhibition caused by renatured His_6_-tagged mature SnOLP, as measured (software UTHSCSA Image Tool, Version 3.00 [57]) in three replicates, similar to the bioassay Petri dishes shown in A and B, for each fungus/oomycete and for each dose/concentration of SnOLP separately. Standard deviation bars are shown for each average column. The averages were statistically compared by using ANOVA and Tukey Test at the probability level of 1% (software Genes [58]). Different letters above the average columns (i.e. a, b and c), indicate that the average values were considered to be statistically different among each other, whereas statistically identical average values are indicated by the same letter. **C**. *Phytophthora nicotiana *var. *parasitica*; 1 day after adding protein (a.a.p.); **D**. *Macrophomina phaseolina*, 5 days a.a.p. ; **E**. *Colletotrichum gloeosporioides*, 12 days a.a.p.; **F**. *Colletotrichum gossypii *var. *cephalosporioides*, 5 days a.a.p.; **G**. *Fusarium solani *f. sp. *glycines*, 5 days a.a.p.

The activity of the refolded His_6_-tagged mature SnOLP was determined by *in vitro *inhibition of mycelial growth of one plant oomycete (*Phytophthora nicotiana *var. *parasitica*) and four plant fungi (*Fusarium solani *f. sp. *glycines*, *Macrophomina phaseolina*, *Colletotrichum gloesporioides*,*Colletotrichum gossypii *var. *cephalosporioides*). The tested concentrations of refolded His_6_-tagged mature SnOLP were 0,1 μg/μL, 0,2 μg/μL and 0,3 μg/μL, corresponding to total doses of 1, 2 and 3 μg of SnOLP, respectively (Fig. 3). A dose of 10 μg BSA (negative control) at the concentration of 1 μg/μL had no effect on mycelial growth of any fungi (Fig. 3B and data not shown) or oomycete (Fig. 3A), as expected. On the other hand, a dose of 2000 U of the pharmaceutical fungicide Nistatin (positive control), at the concentration of 200 U/μL, inhibited the mycelial growth of all four fungi (Fig. 3B and data not shown) tested but not of the oomycete (Fig. 3A), also as expected. The highest concentration tested to achieve the maximum inhibitory activity of SnOLP against the four fungi and the oomycete was 0,3 μg/μL (Fig. 3). The sensitivity of almost all pathogens to SnOLP increased at growing concentrations of the protein (Fig. 3C, D, E, G), except for *Colletotrichum gossypii *var. *cephalosporioides*, which was equally sensitive to SnOLP at both 0,2 and 0,3 μg/μL concentrations (Fig. 3F). *Macrophomina phaseolina *was the only pathogen tested which was not sensitive to SnOLP at the concentration 0,1 μg/μL (Fig. 3B, D). In summary, these results prove that SnOLP directly inhibits growth of the pathogens tested, in a dose and concentration dependent manner.

## Discussion

In this work, we report the successful expression, purification, refolding and antimicrobial activity of the neutral His_6_-tagged mature SnOLP, a *Solanum nigrum *var. *americanum *osmotin belonging to the antimicrobial PR-5 protein family. The results demonstrated that SnOLP is effective against several agronomically important plant pathogens. SnOLP, as well as other neutral and basic members of the PR-5 protein family, require the formation of eight disulfide bonds for its biological activity and are synthesized in plants as an inactive precursor (i.e. preproprotein). In general, the preproprotein precursor contains an N-terminal signal peptide, which mediates the transport of the protein through the secretory pathway [33,34], and an additional carboxy-terminal extension, which may be removed during or after transport to the plant vacuole [29,34,35]. Although bacterial expression systems neither perform certain post-translational modifications (such as removal of signal peptides) nor form all disulfide bonds of eukaryotic proteins correctly, it is still a faster and cheaper system for heterologous expression than other eukaryotic cell systems, such a yeast, insect or mammalian cells. Therefore, we chose to adapt an *E. coli *expressing system coupled with *in vitro *post-expression refolding to successfully produce high amounts of active forms of SnOLP.

In order to express mature SnOLP in *E. coli*, the signal peptide and the carboxy-terminal extension from the prepro *SnOLP *ORF [32] was deleted by PCR-engineering, and the resulting PCR-fragment was cloned into the bacterial expression vector pQE30, which encodes a His_6_-fusion tag. The resulting pQE30-SnOLP construct was introduced into *E. coli *for the expression of His_6_-tagged mature SnOLP. The conditions herein established to overexpress His_6_-tagged mature SnOLP in *E. coli *(induction with 0.4 mM IPTG and incubation at 37°C for 3 h) led to formation of protein inclusion bodies, abundantly present within the insoluble protein fraction. This appears to be frequently observed when proteins are overexpressed and exceed around 30% of the total host cell protein contents. Inclusion bodies, amyloids and protein precipitation are common manifestations of protein aggregation, in which misfolded protein molecules may be present [36]. However, the probable misfolding of the insoluble His_6_-tagged mature SnOLP overexpressed in *E. coli *was successfully repaired by denaturing and subsequent refolding procedures. These corrective procedures resulted in biologically active conformations of the recovered mature SnOLP that, according to results herein shown, exerted antimicrobial action towards plant pathogenic fungi and oomycete. Despite the amount of correctly refolded mature SnOLP was not quantified, the use of reduced:oxidized gluthatione redox buffer, which can produce a mixture of correctly refolded and misfolded protein, folding intermediates and kinetic traps [37], yielded biologically functional SnOLP.

A small number of PR5-like proteins have also been successfully expressed in *E. coli *in a biologically active form [31,13]. Hu and Reddy [31] demonstrated that an *Arabidopsis *thaumatin-like protein (ATLP3) could be expressed in *E. coli *in form of inclusion bodies, purified and that the refolded mature form displayed activity against some pathogenic fungi. Newton and Duman [13] found that an osmotin-like cryoprotective protein from *Solanum dulcamara*, when expressed in *E. coli *and directed to periplasmic localization, resulted in high concentrations of the soluble protein with cryoprotective activity, whereas when it was expressed in the bacterial cytoplasm, high amounts of insoluble and aggregated proteins were produced.

Here, we demonstrated that the SnOLP protein, in a refolded fusion-mature form, displays activity against a spectrum of four fungi and one oomycete, which were chosen due to their economical importance as plant pathogens. Sudden death syndrome of soybean (*Glycine max *(L.) Merr.), caused by several species of *Fusarium *belonging to the taxonomic section Martiella, among them *F. solani *f. sp. *glycines*, is a disease of increasing economic importance in soybean producing countries, such as Brazil [38-40]. Moreover, *Macrophomina phaseolina*, which causes the charcoal rot disease in soybean root and stem, has been considered one of the most prevalent soybean pathogens in Brazil [41,42]. Furthermore, *Colletotrichum gossypii *var. *cephalosporioides *is cited as the etiological agent of ramulose, one of the most impacting fungal diseases occurring on cotton (*Gossypium hirsutum *L. var. *latifolium *Hutch) in Brazil, which provokes super budding of the plant flushing tissues [43]. *Phytophthora nicotiana *var. *parasitica *causes root rot and gummosis in citrus (*Citrus *spp.) worldwide, especially in rootstock plants, leading to serious damage and losses in citrus seedbeds, nurseries, as well as young and mature groves [44-46]. Finally, *Colletotrichum gloesporioides *causes anthracnoses, a relevant disease occurring on a wide range of plants, including *Stylosanthes guianensis*, an important tropical forage plant [47,48]. Therefore, the discovery of a protein, such as SnOLP, that affects the growth of these pathogens could be of uppermost relevance for the transgenic control of these diseases in economically important crops.

As shown in Figure 3, the sensitivity of the pathogens to SnOLP appears to be species specific, since different levels of sensitivity were observed among unrelated pathogen species. Nevertheless, all the tested pathogens were sensitive to 2 and 3 μg of refolded His_6_-tagged mature SnOLP. Abad et al. [15] demonstrated that 30, 60 e 100 μg of a native tobacco osmotin had no effect against *Macrophomina phaseolina*. Likewise, in the presence of 100 μg of the referred tobacco protein, the mycelial growth of *Colletotrichum gloesporioides *was slightly inhibited. The mycelial growth of *Fusarium *spp. was considered only weakly inhibited, and also the inhibition of *Phytophthora *spp. was visible as a discrete zone beyond the protein disc [15]. Our results demonstrate that SnOLP exerts *in vitro *inhibitory effect upon mycelial growth of the tested pathogens, though some of these pathogens are not sensitive to the before mentioned tobacco osmotin [15].

Another osmotin isolated from *Solanum nigrum*, denoted as SniOLP and which is 99% identical to SnOLP, when expressed in *E. coli *and subsequently refolded, exerted antifungal activity against *Rhizoctonia batiticola *and *Sclerotinia sclerotiorum *[49]. Therefore, it is very likely that SnOLP also presents activity against these fungi. Some osmotins present endo-β-1,3-glucanase activity, which is speculated to be involved in the antifungal mechanism of action of osmotins [50,51]. Similarly to SniOLP, which does not present endo-β-1,3-glucanase activity, SnOLP most like does not have glucanase activity as well.

Our results are relevant from a biological point of view, since the *SnOLP *gene was isolated from a solanaceous weed, *Solanum nigrum *var. *americanum*, common in the Americas. It was reported that a quite similar species, an European *S. nigrum *variety, is a nonhost plant possessing resistance to *Phytophthora infestans*, a destructive oomycete causing late blight disease in potato [24]. In addition, it was demonstrated that the penetration of *S. nigrum *leaf epidermis by *P. infestans *was accompanied by rapid Hypersensitive Response of plant cells, within 22 h after inoculation, what resulted in abortion of the infection [52,53]. Evidences pointed out that the constitutive expression of PR genes, including PR-5, may contribute to non-specific host resistance to *P. infestans *[26] and that PR-5 proteins are induced in potato in response to infection by this pathogen [7,54]. However, no indication for a participation of PR-5 proteins in nonhost resistance response was given so far. Here we provided one more evidence for the specific antioomycetal effect of PR-5 proteins targeted to *Phytophthora *spp. Despite of the lack of ultimate evidence, we hypothesize that the *SnOLP *gene might also be involved in basal defense responses to oomycetes and/or fungi in *Solanum nigrum*. Further analysis will contribute to elucidate a possible biological function or involvement of the *SnOLP *gene in defense responses of *S. nigrum *to pathogens.

## Conclusion

We report here the successful overexpression in *E. coli*, purification, refolding and antifungal activity of the SnOLP osmotin from *S. nigrum*. The herein demonstrated inhibition of *in vitro *mycelial growth of economically important pathogens of soybean, cotton and citrus by SnOLP reveals promising features for biotechnological applications, being this is a subject of our current researches.

## Methods

### Construction of expression vector, cloning and transformation of *E. coli*

The isolation of *SnOLP *gene and used primers were described previously by Campos et al. [32]. The complete open reading frame (ORF) sequence coding for the wild type preproprotein SnOLP (GenBank accession no AF450276) was amplified by PCR from black nightshade(*Solanum nigrum *var. *americanum*) genomic DNA [32]. This ORF was used as template to prepare the prepro-truncated form (Figure 1). The prepro-truncated form, coding for mature SnOLP protein, was obtained by usage of the primers PPM1 (CGCGGATCCGCTGCGACTATCGAGGTACGC), containing a suitable *Bam*H I cloning site (underlined within the primer sequence), and PPM2 (CCCAAGCTTACCCTTAGGACAAAAGACAACCC), containing a *Hind *III site (underlined within the primer sequence), and high fidelity *Pfu *DNA polymerase (Invitrogen).

The PCR amplified fragments were eluted from agarose gel, cloned into pGEM-T Easy vector (Promega) and subcloned into the *Bam*H I and *Hind *III sites of the expression vector pQE30 (QiaExpressionist-QiaGen), which contains a N-terminal His_6_-tag extension, resulting in the pQE30-SnOLP construct. The recombinant clones were selected by using 5-bromo-4-chloro-3-indolyl-β-D galatopyranoside (X-Gal)/Isopropyl-β-D-thiogalactopyranoside (IPTG) blue/white colony screening system and digested with appropriated restriction enzymes. Thereafter, the presence of correct deletion mutations and the correct frame were verified by sequencing entirely both strands of the coding region of His_6_-tagged mature SnOLP, by using an automated ABI sequencer with BigDye terminator cycle sequencing kit (Perkim-Elmer). Sequences were analyzed by using the UWGCG software Package (Version 9.1 Genetics Computer Group, Wisconsin, Madison, Wisc.). The pQE30-SnOLP expression construct containing inserts coding for His_6_-tagged mature SnOLP was introduced into *E. coli *M15 strain (QiaGen) competent cells using standard transformation techniques.

### Overexpression of His_6_-tagged mature SnOLP in *E. coli*

Preliminary experiments with the pQE30-SnOLP expression construct were performed to determine the solubility of the SnOLP, according to the instructions of manufactures (QiaEXpressionist-QiaGen), and to establish the expression levels of the mature protein. Cells of *E. coli *M15 strain carrying out the construct were cultured overnight at 37°C in 5 mL of Luria-Bertani (LB) medium containing 100 μg/mL ampicillin and 25 μg/mL kanamycin (i.e. LB selective medium) under vigorous agitation (200 rpm). This pre-inoculum suspension was then used to inoculate 500 mL fresh LB selection medium, which was agitated until an O.D. 600 of 0.6 was reached. Thereafter, an aliquot of non induced control cells was colleted and reserved, and the expression of His_6_-tagged mature SnOLP protein was induced in the left cells by addition of IPTG to a final concentration of 0.4 mM. The cells were cultivated at 37°C for 3 h in the induction medium (i.e. LB selective medium plus IPTG) and afterwards collected by centrifugation at 4.000 *g *at 4°C for 20 min.

### Purification of the His_6_-tagged mature SnOLP protein

As an attempt to determine the solubility of the protein, an aliquot of soluble fraction was recovered after centrifugation of cellular lysate, obtained from an aliquot of induced pelleted cells by using lysis buffer (50 mM potassium phosphate buffer pH 7.8, 400 mM NaCl, 100 mM KCl, 0.5% Triton X-100, 10% glycerol, 10 mM Imidazole), under native conditions.

For a larger scale experiment, the pelleted cells were resuspended in denaturing lysis buffer (0.1 M Tris.HCl, 6 M Urea, pH 8.0) and the cellular suspension was maintained under gentle shaking for 1 hour at room temperature. Cellular debris were removed by centrifugation at 10.000 *g *for 25 min at 4°C in order to obtain the solubilized fraction from the supernatant. Final purification of His_6_-tagged mature SnOLP was performed by high-performance immobilized-metal ion affinity chromatography (IMAC) on 10 mL batches of 50% Nickel-nitrilotriacetic-acid (Ni-NTA) resins (QiaEXpressionist-QiaGen), under constant shaking for 1 hour. In order to eliminate proteins nonspecifically bound to the column, the resin was submitted to the washing solution (0.1 M Tris.HCl, 6 M Urea, 20 mM Imidazol, pH 8.0) and afterwards the recombinant protein was eluted in two steps by using 10 mL of 0.1 M Tris.HCl, 6 M Urea, 200 mM Imidazol, pH 8.0. The eluted fractions were collected and reserved until use.

### Protein gel and Western blotting analysis

Protein expression and purification were monitored by sodium dodecyl sulfate-polyacrilamide gel electrophoresis (SDS-PAGE) [55]. Typically, an aliquot of 50 μl of soluble and insoluble fractions were mixed with loading buffer (1:1) consisting of 250 mM Tris-HCl pH 6.8, 150 mM dithiothreitol, 35% (w/v) glycerol, 7% SDS and 0.2% (w/v) bromophenol blue, and boiled for 5 min. Protein samples were electrophoresed on 12% SDS-PAGE gels and visualized by Comassie Blue staining (10% (v/v) methanol, 10% (v/v) acetic acid and 0.0125% (w/v) Comassie G-250). The distaining solution was 10% (v/v) methanol and 10% (v/v) acetic acid in water.

For Western blotting, following SDS-PAGE, the protein samples were transferred onto nitrocellulose Hybond membranes (Amersham Pharmacia) using a semi-dry blotting cell (BioRad). The membranes were blocked and incubated with His_6 _monoclonal antibody (Clontech), according to the instructions of manufacturer (1:5000 dilutions) for 60 min. After washing the membranes, the blot was incubated with anti-mouse IgG secondary antibody conjugated to alkaline phosphatase (Sigma) (1:5000 dilution) for 60 min. Immunoreactive bands were detected colorimetrically by immersing the blot into alkaline phosphatase substrate solution (0.3 mg/ml nitro blue tetrazolium (NBT) and 0.15 mg/ml 5-bromo-4-chloro-3-inodolyl phosphate (BCIP) in 0.1 M Tris pH 9.5, 0.1 M NaCl, 50 mM MgCl_2_).

### Refolding of recombinant SnOLP

Renaturation of solubilized mature His_6_-SnOLP was performed by dropwise mixing purified SnOLP protein under denaturing conditions into refolding buffer (20 mM Tris.HCl pH 7.5, 500 mM NaCl, 10 mM reduced glutathione, 1 mM oxidized glutathione, and 20% (w/v) glycerol), at 4°C, under stirring, in a protein:buffer ratio of 1:10. After repeated dialysis processes, at 4°C, performed with deionized water to slowly remove denaturants, the recombinant protein was concentrated by lyophilization. Lyophilized recombinant SnOLP was resuspended in water for use in *in vitro *bioassays. Protein concentration was determined spectrophotometrically by the method of the Bradford [56], using Bio Rad Protein Assay with Bovine Serum Albumin (BSA) as the standard.

### Fungal growth inhibition bioassays

The purified and refolded recombinant SnOLP protein was assayed for its ability to inhibit the *in vitro *mycelial growth of plant pathogenic oomycete and fungi, essentially as described by Abad *et al*. [15]. The pathogens used for the tests were *Macrophomina phaseolina *(Tassi) Goidanich and *Fusarium solani *(Mart.) f. sp.*glycines *isolates from *Glycine max *(soybean), *Colletotrichum gloesporioides *(Penz.) Penz & Sacc. isolate from *Stylosanthes guianensis*,*Colletotrichum gossypii *South. var.*cephalosporioides *Costa isolate from *Gossypium hirsutum *(cotton), and *Phytophthora nicotiana *(Breda de Haan) var.*parasitica *(Dast.) Waterh isolate from *Citrus *sp. All fungi were cultivated in PDA (potato dextrose agar, Sigma) and the oomycete *P. nicotiana *var. *parasitica *was grown in CA (carrot agar) from mycelial disc placed at the center of agar plates, for up to three days. Then, sterile paper discs were positioned adjacent to the growing colony margin and saturated with 10 μL of either BSA 10 μg (Sigma) as a negative control, or Nistatin (Micostatin 2000 U; generic pharmaceutical) as a positive control, or of recombinant refolded SnOLP protein to different doses (1, 2 or 3 μg). The plates were further incubated and daily monitored for up to twelve days. Experiments were conducted with three replicates, digitally photographed and the area of mycelial growth inhibition was measured (Free software: UTHSCSA Image Tool, Version 3.00) [57] for each pathogen and for each concentration of SnOLP separately. Statistical analyses were performed to compare the average areas of mycelial growth inhibition by using ANOVA and Tukey Test at the probability level of 1% (Software: Genes) [58].

## Competing interests

The author(s) declare that they have no competing interests.

## Authors' contributions

MAC: She carried out the cloning, expression and purification of SnOLP. Moreover, she helped MSS to analyze the bioassays and drafted the manuscript. MSS: She carried out the bioassays and the analyzes related to them. Moreover, she drafted the manuscript. CPM: He carried out the refolding of SnOLP and helped out MSS on the bioassays. SGR: She carried out the cloning, expression and purification of SnOLP together with MAC. RPDS: He carried out the refolding of SnOLP together with CPM and helped out MSS on the bioassays. EAV: He carried out all the statistical analyzes of the bioassays and drafted the manuscript. MFGS: She is the senior researcher and leader of the present group. She has been involved in drafting the manuscript and revising it critically for important intellectual content. Moreover, she has given final approval of the version to be published.
